# Magnet-assisted endoscopic removal of ingested sewing needles from the stomach and descending duodenum

**DOI:** 10.1055/a-2210-0248

**Published:** 2023-12-12

**Authors:** Qi Luo, Yang Li, Liansong Ye, Lifan Zhang, Meiting Liang, Bing Hu, Yi Mou

**Affiliations:** 1 34753 Department of Gastroenterology and Hepatology, Sichuan University West China Hospital, Chengdu, China; 2 34753 Division of Gastrointestinal Surgery, Department of General Surgery, Sichuan University West China Hospital, Chengdu, China

A 42-year-old man presented to our emergency department with a history of having swallowed several sewing needles an hour previously. Physical examination showed mild hyperemia of the pharynx and slight abdominal tenderness. Cervical thoracoabdominal computed tomography detected several high density shadows in the stomach and duodenum, without any signs of perforation ( Fig. 1 ). Gastroscopy revealed the presence of three sharp-pointed sewing needles in the stomach and two in the descending duodenum ( Fig. 2 ). The lengths of the needles were approximately 3–4 cm. Neither foreign forceps nor snares were able to grasp the needles tightly owing to their uncontrollable slim shape.

**Fig. 1:**
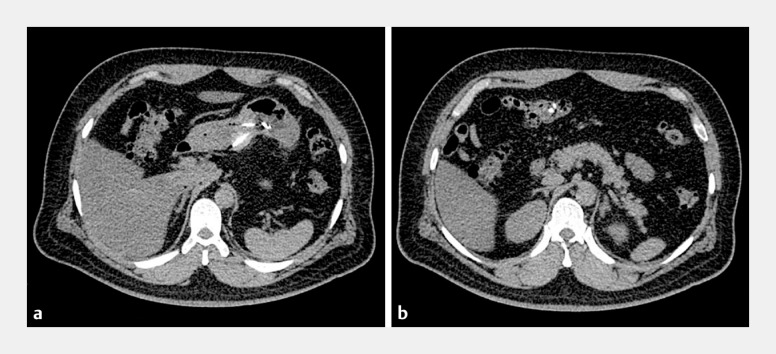
Cervical thoracoabdominal computed tomography images showing several high density shadows: **a** in the stomach; **b** in the duodenum.

**Fig. 2:**
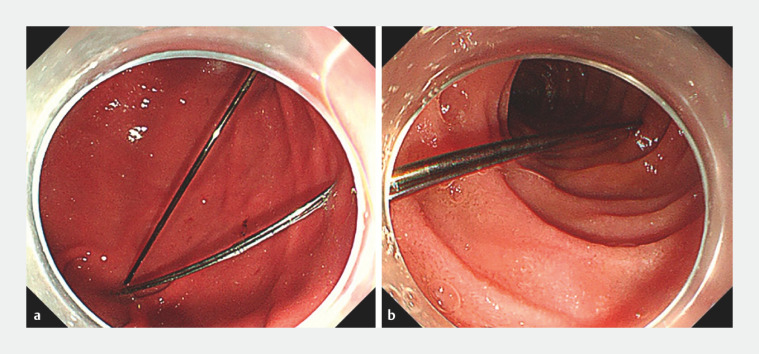
Gastroscopic views showing: **a** three sewing needles in the stomach cavity; **b** two sewing needles in the descending duodenum.

Given that the needles are metal, a magnet grasped by forceps was subsequently used to retrieve them ( Video 1 ). The five sewing needles were held by the magnet throughout the removal process and successfully extracted ( Fig. 3 ). Mild mucosal injury was noted in the stomach and the descending duodenum, without evidence of significant bleeding, obvious perforation, or needle embedment. An abdominal radiograph was performed after the procedure, which confirmed no other needles were present. The patient reported no further discomfort, and was discharged the same day.

Magnet-assisted endoscopic removal of ingested sewing needles from the stomach and descending duodenum.Video 1

**Fig. 3:**
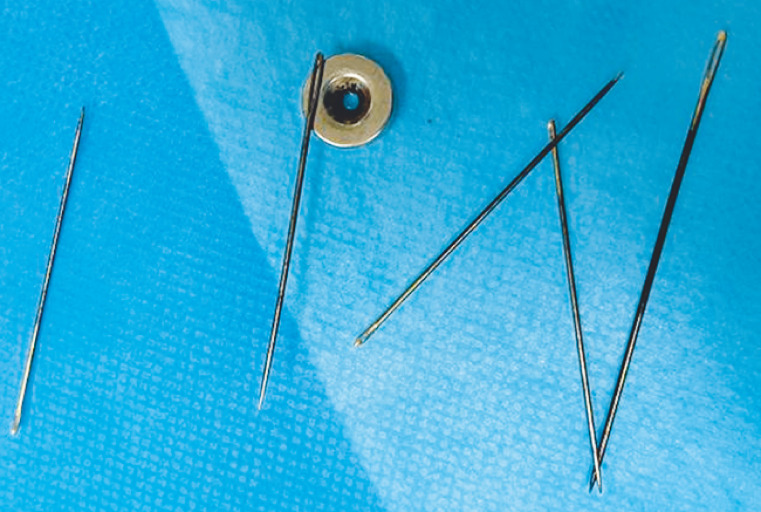
Photograph of the five sewing needles following their successful removal.

The ingestion of sharp-pointed foreign bodies such as sewing needles is an urgent situation, with an increased risk of perforation. Commonly used retrieval devices for sharp-pointed foreign bodies are forceps or snares 1 , but our failed attempts show that these are not efficient for sewing needles. We have previously reported on the wide applications of magnets in various endoscopic therapies 2 3 4 5 , and this case demonstrates that magnets can be an alternative for endoscopic removal of sewing needles.

Endoscopy_UCTN_Code_TTT_1AO_2AL

## Notes

Endoscopy E-Videos https://eref.thieme.de/e-videos
*E-Videos* is an open access online section of the journal *Endoscopy* , reporting on interesting cases and new techniques in gastroenterological endoscopy. All papers include a high-quality video and are published with a Creative Commons CC-BY license. Endoscopy E-Videos qualify for HINARI discounts and waivers and eligibility is automatically checked during the submission process. We grant 100% waivers to articles whose corresponding authors are based in Group A countries and 50% waivers to those who are based in Group B countries as classified by Research4Life (see: https://www.research4life.org/access/eligibility/ ). This section has its own submission website at https://mc.manuscriptcentral.com/e-videos .
